# Reply to comment on “Cardiovascular safety of febuxostat compared to allopurinol for the treatment of gout: A systematic and meta‐analysis”

**DOI:** 10.1002/clc.23852

**Published:** 2022-05-30

**Authors:** Linggen Gao, Bin Wang, Rui Cheng

**Affiliations:** ^1^ Department of Comprehensive Surgery General Hospital of Chinese People's Liberation Army & National Clinical Research Center for Geriatric Disease Beijing China

We thank profs. Deng and Zhang for their comment on our manuscript.^1^

First, profs. Deng and Zhang mentioned that it was unknown why we selected randomized controlled trials (RCTs) and observational studies simultaneously in the same meta‐analysis. A high quality meta‐analysis based on RCTs is regarded as the highest level of evidence‐based medical evidence, and the formulation of many clinical guidelines also follows this standard, but this does not mean that we should unconditionally believe in RCT‐based meta‐analysis, especially when the results of RCTs are different from those of observational efficacy comparison trials. The implementation of RCTs is limited by the scale of funds and usually can only ensure the statistical efficiency of the primary outcome indicators. Considering the operability and other factors, the included population is often different from that in clinical practice, the external applicability of evidence is limited, and the direct application to clinical practice sometimes cannot achieve the expected effect. The population included in the observational efficacy comparison study is closer to clinical practice, but since there is no randomization, there are many factors that may interfere with the final results. When answering a clinical question, do not just believe in RCTs. Observational efficacy comparative studies also need to be included in the evaluation. Moreover, if the results of RCTs are inconsistent with those of observational studies, it is more necessary to carefully evaluate the reasons for the differences. Sometimes the conclusions of observational studies are more credible.^2^ In fact, the current meta‐analysis can integrate the results of observational studies and RCTs at the same time, so as to make full use of the data and get a more comprehensive result.

Second, both profs. Deng and Zhang have noted that two eligible published studies were missed in our meta‐analysis. One of the missed studies compares major cardiovascular events in patients with gout and concurrent cardiovascular disease and chronic kidney disease who receive febuxostat or allopurinol.^3^ One of the exclusion criteria in our meta‐analysis was patients with severe kidney dysfunction. Therefore, one of the studie suggested by profs. Deng and Zhang did not meet the inclusion criteria. The study recommended by the authors was included and the meta‐analysis was updated^4^ (Figure 1). Compared with allopurinol treatment group, the febuxostat group had a better safety outcome, which was the composite of urgent coronary revascularization^1^ (odds ratio [OR]: 0.84, 95% confidence interval [CI]: 0.77–0.90, *p* < .0001 and stroke (Figure 1(A)) (OR: 0.87, 95% CI: 0.79–0.96, *p* = .006). However, that difference was not found in nonfatal myocardial infarction (Figure 1B) (OR: 0.95 95% CI: 0.79–1.15, *p* = .63), cardiovascular‐related mortality (Figure 1C) (OR: 1.01, 95% CI: 0.72–1.43, *p* = .94), and all‐cause mortality (Figure 1D) (OR: 0.97, 95% CI: 0.78–1.22, *p* = .82).

Figure 1Meta‐analysis of studies that compared the safety of febuxostat therapy and allopurinol‐treated patients with gout during follow‐up. (A) Nonfatal stroke; (B) nonfatal myocardial infarction; (C) cardiovascular death; and (D) death from any cause.

**Figure:**
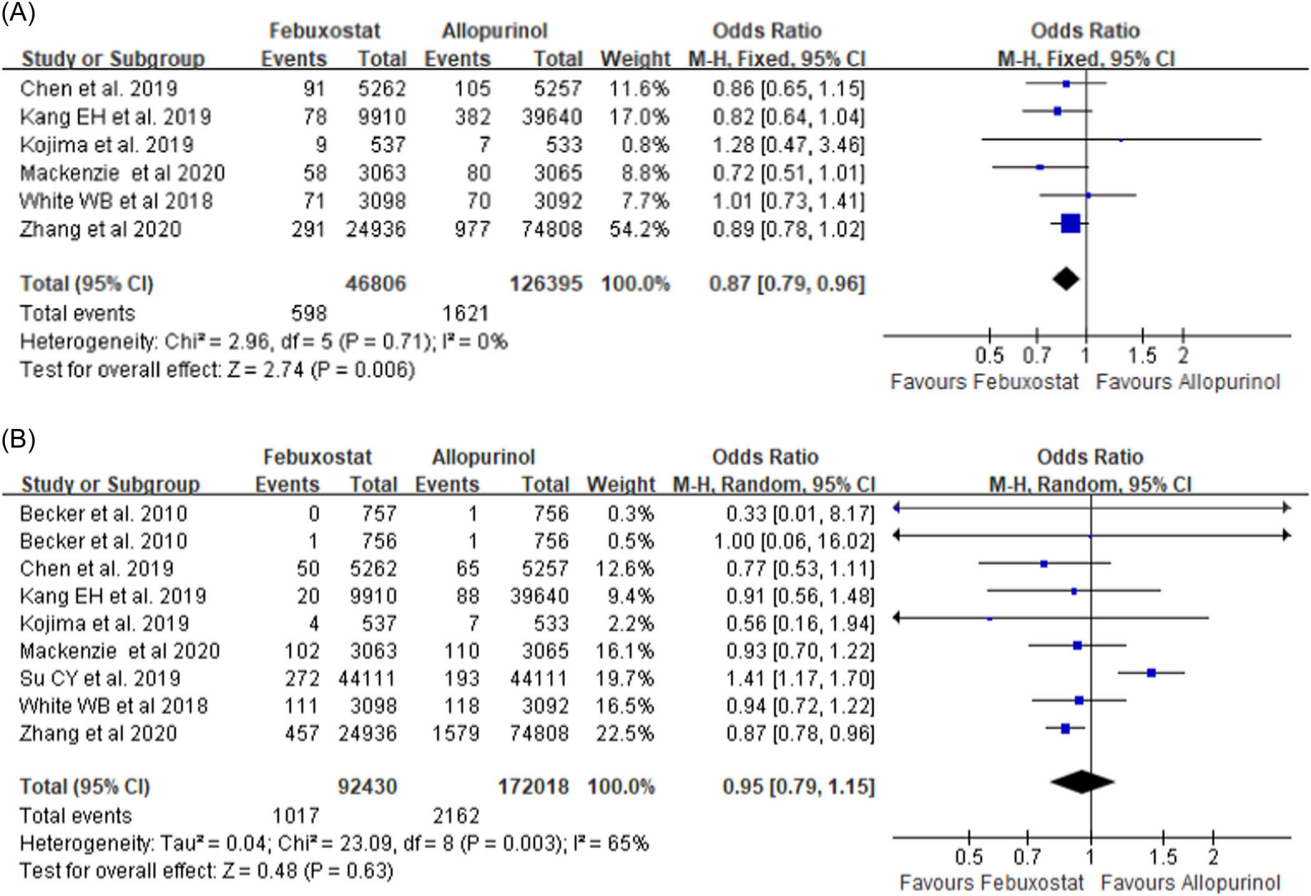

**Figure:**
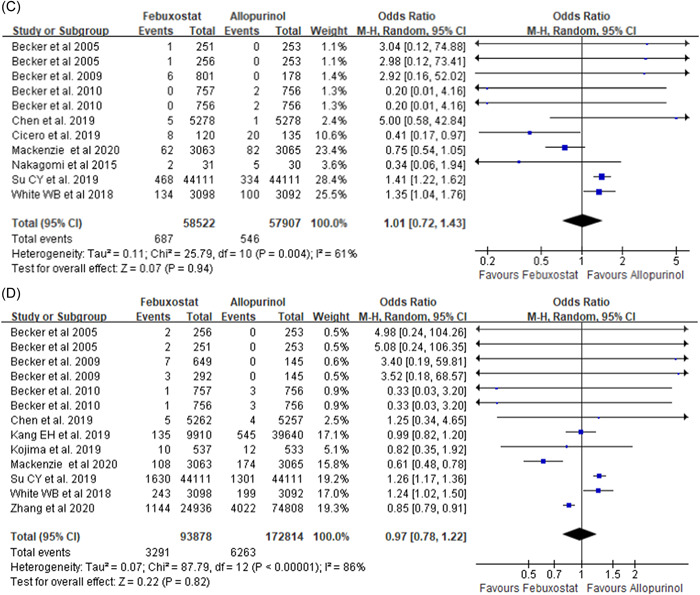

Third, we agree with profs. Deng and Zhang that another probably missing critical point is the quality assessment of the included studies. Study quality was independently assessed (by Gao L. G. and Cheng R.) according to a tool that was specifically developed for the randomized studies based on the Delphi Consensus.^5^ The following criteria were used for scoring the quality of each study: (a) Was a method of randomization performed? (b) Was the treatment allocation concealed? (c) Were the groups similar at baseline regarding the most important prognostic indicators? (d) Were the eligibility criteria specified? (e) Was the outcome assessor blinded? (f) Was the care provider blinded? (g) Was the patient blinded? (h) Were point estimates and measures of variability presented for the primary outcome measures? (i) Did the analysis include an intention‐to‐treat analysis? A combined quality score was obtained by adding the scores for each criterion. Thus, the quality score could range from 0 to 9 points. Study quality was rated as poor (scores ≤ 3), fair (4–5), or high (>5). All of the included randomized studies were of high quality on the Delphi consensus criteria.

The Newcastle Ottawa Scale (NOS) was used to assess the quality of the included nonrandomised studies.^6^ Using this scale, each study is judged on eight items, categorized into three groups: the selection of the study groups; the comparability of the groups; and how diet pattern was ascertained (objectively or subjectively). Stars are awarded for each quality item and the highest quality studies are awarded up to nine stars. A study is considered of good quality if there are three or four stars in the selection domain and one or two stars in the comparability domain and two or three stars in the outcome/exposure domain. For NOS, a score of at least six out of nine indicated high quality.^7^ All of the included nonrandomized studies were of high quality according to NOS.

We have benefited a lot from this discussion. This meta‐analysis was updated and supplemented according to valuable opinions, which further makes up for the shortcomings of this analysis. We appreciate the authors' comments on our study. Your valuable suggestions will be of great help to our future scientific research work.

## CONFLICT OF INTEREST

The authors declare no conflict of interest.

## References

[1] Gao L , Wang B , Pan Y , Lu Y , Cheng R . Cardiovascular safety of febuxostat compared to allopurinol for the treatment of gout: asystematic and meta‐analysis. Clin Cardiol. 2021;44(7):907‐916. 34013998 10.1002/clc.23643 PMC8259158

[2] Gershon AS , Jafarzadeh SR , Wilson KC , Walkey AJ . Clinical knowledge from observational studies. Everything you wanted to know but were afraid to ask. Am J Respir Crit Care Med. 2018;198(7):859‐867. 29733678 10.1164/rccm.201801-0118PP

[3] Foody J , Turpin RS , Tidwell BA , Lawrence D , Schulman KL . Major cardiovascular events in patients with gout and associated cardiovascular disease or heart failure and chronic kidney disease initiating a xanthine oxidase inhibitor. Am Health Drug Benefits. 2017;10(8):393‐401. 29263773 PMC5726059

[4] Chen CH , Chen CB , Chang CJ , et al. Hypersensitivity and cardiovascular risks related to allopurinol and febuxostat therapy in Asians: a population‐based cohort study and meta‐analysis. Clin Pharmacol Ther. 2019;106(2):391‐401. 30690722 10.1002/cpt.1377

[5] Verhagen AP , de Vet HC , de Bie RA , et al. The Delphi list: a criteria list for quality assessment of randomized clinical trials for conducting systematic reviews developed by Delphi consensus. J Clin Epidemiol. 1998;51(12):1235‐1241. 10086815 10.1016/s0895-4356(98)00131-0

[6] Wells GASB , O'Connell D , Peterson J , Welch V , Losos M , Tugwell P . The Newcastle‐Ottawa Scale (NOS) for assessing the quality of nonrandomised studies in meta‐analyses; 2016. https://www.ohri.ca/programs/clinical_epidemiology/oxford.asp

[7] Athanasiadis DI , Martin A , Kapsampelis P , Monfared S , Stefanidis D . Factors associated with weight regain post‐bariatric surgery: a systematic review. Surg Endosc. 2021;35(8):4069‐4084. 33650001 10.1007/s00464-021-08329-w

